# Comparative Diagnostic Efficacy of Ultrasonography and Radiography for Gas Embolism in Loggerhead (*Caretta caretta*) Turtles

**DOI:** 10.3390/ani14243623

**Published:** 2024-12-16

**Authors:** Carmela Valastro, Delia Franchini, Stefano Ciccarelli, Serena Paci, Daniela Freggi, Diego Boscia, Pasquale Salvemini, Antonio Di Bello

**Affiliations:** 1 Department of Veterinary Medicine, University of Bari “Aldo Moro”, Strada Provinciale 62 per Casamassima Km 3, 70010 Valenzano, Italy; delia.franchini@uniba.it (D.F.); stefano.ciccarelli@uniba.it (S.C.); serena.paci@uniba.it (S.P.); daniela.freggi@uniba.it (D.F.); diego.boscia@uniba.it (D.B.); antonio.dibello@uniba.it (A.D.B.); 2 WWF Molfetta Rescue Center, Via Puccini 16, 70056 Molfetta, Italy; crtmolfetta@gmail.com

**Keywords:** ultrasonography, radiography, loggerheads, gas embolism

## Abstract

Sea turtles face numerous threats, often stemming from human activities, resulting in high mortality rates. One of the primary risks they encounter is posed by fishing activities. In the South Adriatic Sea, the extensive trawling fleet often impacts sea turtles, and in recent years, a specific disorder, known as gas embolism (GE), and the associated disease known as decompression sickness (DCS), has emerged as a new threat. Our study aims to compare the statistical concordance and sensitivity, specificity, and accuracy between ultrasonography and radiography for evaluating GE in marine turtles. The study involved the analysis of 29 loggerhead turtles (*Caretta caretta*) admitted to the Sea Turtle Clinic (STC) at the Department of Veterinary Medicine, University of Bari, Italy, between December 2022 and March 2023. The sea turtles underwent X-ray evaluation using the three standard projections (dorso-ventral, latero-lateral, cranial-caudal), followed by ultrasound examination to visualize blood vessels through cervical, axillary, and inguinal ultrasound windows. Color Doppler ultrasonography was utilized to assess blood flow, gas localization, and quantity, but this technique proved to be less helpful in detecting GE. Our results confirm the statistically valid performance of ultrasonographic examinations, highlighting the significant role of combining ultrasonography and radiography to enhance sensitivity, especially in complex and challenging cases for identifying gas embolism (GE) in sea turtles.

## Simple Summary

Sea turtles face numerous threats, many of which are linked to human activities like fishing. In the South Adriatic Sea, trawling poses a major risk, and a new threat has emerged: gas embolism (GE). This condition is increasingly diagnosed in sea turtles exposed to sudden pressure changes during fishing operations. This study compares the effectiveness of ultrasonography and radiography in diagnosing GE in sea turtles. Examinations were conducted on loggerhead turtles admitted to the Sea Turtle Clinic at the University of Bari, Italy. The turtles underwent X-ray and ultrasound exams, where blood vessels were examined using cervical, axillary, and inguinal windows. Although Color Doppler ultrasonography was less effective for detecting GE, the combined use of ultrasonography and radiography improved sensitivity and diagnostic accuracy. These findings emphasize the importance of integrating both techniques for more reliable detection of GE in marine turtles, particularly in challenging cases.

## 1. Introduction

In sea turtles unintentionally caught by trawlers, pulmonary edema, emphysema, contusions, pneumonia can be frequently observed [1,2]. These conditions may inhibit diving, compromising their survival capabilities [3,4]. In 2014, a study of bycaught loggerhead sea turtles (*Caretta caretta*) entrapped at depth in trawls and gillnets demonstrated that sea turtles may develop gas embolism (GE) and the consequent disorder known as decompression sickness (DCS) [5]. This condition might be induced by a sudden drop in external pressure [6], causing the transition of gases in tissues from dissolved to gaseous states and the subsequent formation of emboli within the turtles’ vessels [7].

The gases responsible for emboli formation include nitrogen and helium. Unlike oxygen (O_2_) and carbon dioxide (CO_2_), these gases cannot be metabolized and instead accumulate within the vascular system [8]. While the exact pathogenic mechanisms of gas embolism in sea turtles remain unclear, it is likely that the emboli pass through the systemic arteries and intrapulmonary arteriovenous anastomoses, leading to systemic gas embolism [9].

In order to detect this condition, a range of diagnostic imaging techniques, including X-rays, CT, and MRI scans, are available [10,11,12,13]. Additionally, the use of Color Doppler ultrasound and echocardiography often proves to be valuable for detecting venous gas emboli [14,15].

A prior study conducted on 128 sea turtles, captured by gillnets or trawls, revealed that 55% of those animals were diagnosed with GE [16]. The diagnosis was established through radiographic examination employing latero-lateral (LL), dorsal-ventral (DV), and cranial-caudal (Cr-Cd) projections, in addition to ultrasound exams.

In our study, we aimed to assess the potential of ultrasonography as a practical alternative to radiographic examination, particularly considering the convenience of conducting this test aboard fishing vessels. We compared the statistical concordance and sensitivity, specificity, and accuracy between ultrasonography and radiography in evaluating GE. Additionally, we verified the utility of Color Doppler ultrasonography in evaluating blood flow. 

## 2. Materials and Methods

The study was conducted on a group of 29 loggerheads, which were admitted to the STC at the Department of Veterinary Medicine in Bari, Italy, within 12–24 h of their capture, between December 2022 and March 2023.

Upon their arrival at the STC, comprehensive medical records were compiled. A thorough clinical examination was performed, assessing the turtles’ nutritional status, muscle tone, sensorium, behavioral attitudes, the condition of their mucous membrane, any pathological observations, and the measurement of body temperature using a digital thermometer deeply inserted into the cloaca. Morphometric measurements were taken: the Curve Carapace Length (CCL), the Curve Carapace Width (CCW), as well as weight measurements.

Before proceeding with diagnostic procedures, any epibionts, epiphytes, and external parasites were removed to prevent interference with imaging processes.

To minimize stress, patients had their vision obstructed using a dark cotton sock or self-fix bandage. The radiographic examination consisted of a complete assessment of the entire body, covering the dorsal-ventral (DV) projection with a vertical beam, and the latero-lateral (LL) and the cranial-caudal (Cr-Cd) projections with a horizontal beam. The DV projections were performed using an X-ray Eurocolumbs ARX 125 KV/300 mA, while the LL and Cr-Cd projections were conducted with the assistance of an X-ray Orange 1060HF Ultra 100 Plus.

For the DV projections, the exposure parameters were configured at KV 70 and mAs 10. Given the variable sizes of the patients, multiple X-rays were taken consecutively to ensure a comprehensive view of the entire body. For the LL and Cr-Cd projection, the exposure parameters were set within a KV range of 65–70 and mAs 12.5. The specific KV value within this range was adjusted based on the size of the animals.

The radiographic images underwent through evaluation by a skilled operator. In cases where signs of GE were identified, the severity was categorized as mild, moderate, or severe following Garcia-Parraga’s classification in 2014 [5]. This classification was contingent on the presence and extent of the embolism observed in both central and peripheral venous and arterial vessels, as well as within the cardiac chambers. The following vascular districts were scrutinized: the precava vein, atria, aorta, sinus venosus, pulmonary vessels, brachiocephalic trunk, hepatic vessels, gastric vessels, postcava vein, inferior mesenteric artery, marginocostal vessels, abdominal veins, abdominal transverse vein, external iliac vessels, renal vessels.

The ultrasound assessments were performed with a portable US device (MyLab Alpha, Esaote, Genova, Italy), by an experienced operator who was blinded to the patient’s clinical condition and the results of the radiographic evaluations.

Among the ultrasound probes, we considered the 2.5 MHz broadband multi-frequency linear probe for the initial trials but, not satisfied with its performance, we preferred to use the broadband multi-frequency Microconvex probe with a 7.5 MHz frequency.

During the ultrasound examination, the animals were positioned in a dorsal recumbent posture, with their carapace resting on a polyester-covered foam rubber mattress, aimed at preventing any harm to the subjects.

To ensure a standardized approach to the technique and its consistent application among animals, the examinations were conducted using the acoustic windows in a fixed order. The procedure commenced with the left prefemoral acoustic window, followed by the right prefemoral, the right axillary, and the left axillary windows, concluding with the ventral cervical window.

Ultrasound examinations were conducted to access the vessels by the following acoustic windows: right and left prefemoral regions for the investigation of hepatic vessels, iliac artery/vein, and renal vessels; right and left axillary regions for a view of the brachial artery and the aortic arch; ventral cervical region for observations of the subclavian artery, aortic arch, and the heart. 

The ultrasound assessments evaluated blood flow in both 2D and Color-Doppler modes.

### Statistical Analysis

To assess the effectiveness of ultrasonography, we utilized the radiographic examination as a gold standard test, a method widely recognized in the literature for diagnosing GE.

Subsequently, we computed the sensitivity index, specificity index, and accuracy.

Next, we ascertained whether the concordance between ultrasonography and radiography was incidental or not through Cohen’s kappa index. This statistical measure evaluates the degree of concordance between tests conducted on the same group of subjects, utilizing data from both radiography and ultrasonography as obtained from the contingency table realized for that purpose.

## 3. Results

A total of 29 turtles were included in this study. The subjects had sizes ranging from 28.1 to 75.9 cm in CCL (mean 65.5 ± 9.6) and from 26.5 to 69.5 cm in CCW (mean 59.5 ± 8.5), and their weight ranged from 2.7 to 59.5 kg (mean 35.0 ± 12.8) (Table S1).

Based on the results of radiographic investigations aimed at detecting signs of GE, the turtles were categorized into groups according to the presence and extent of gas observed in the examined anatomical regions [5].

**Figure 1:**
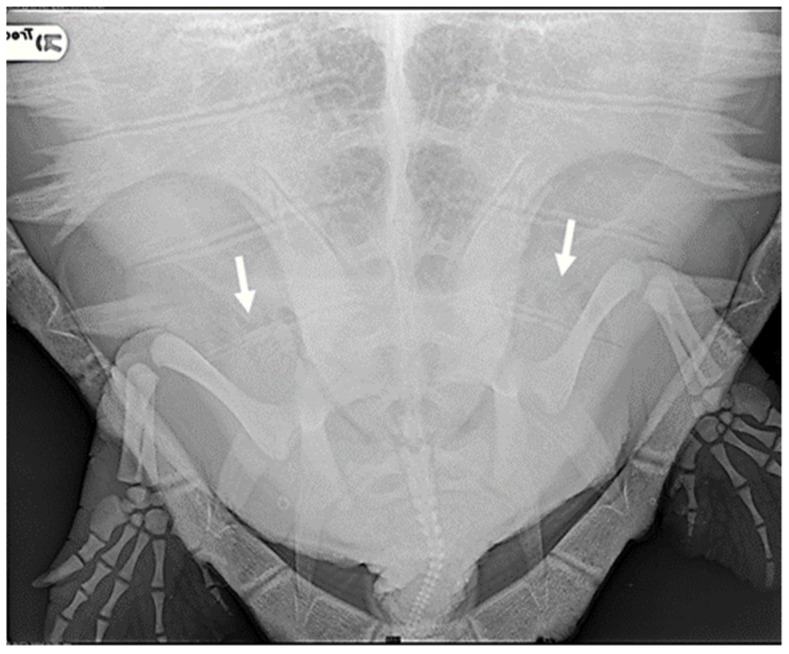
R-ray examination in DV projection of a loggerhead turtle affected by mild GE. It is possible to detect a small amount of gas within the renal vessels (indicated by white arrows).

Specifically, eight animals were classified as negative (27.6%): they exhibited no radiographic signs of gas presence in any of the considered districts. In the case of 11 turtles, gas was observed in only a few anatomical areas, primarily in the marginal-costal vessels and/or renal vessels, leading to their classification as mild cases (37.9%) (Figure 1).

**Figure 2:**
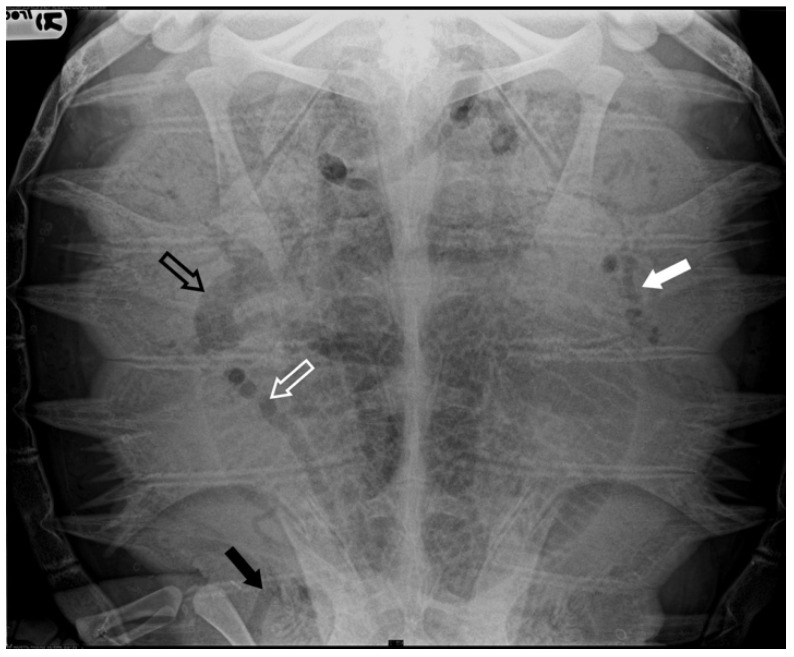
X-ray examination in DV projection of a subject diagnosed with moderate severity GE reveals the presence of gas in various vessels, including the gastric vessels (white arrow), inferior mesenteric artery (white empty arrow), iliac vessels (black arrow), and duodenal vein (black empty arrow). The present gas overlaps the lungs’ cranial area, reducing the visualization of lung volume.

Four subjects were classified as having moderate severity of GE (13.8%): in addition to the renal and marginal-costal vessels, a notable distribution of gas was identified in several other vascular districts, including hepatic vessels, inferior mesenteric artery, gastric artery, abdominal transverse vein, iliac arteries, and veins (Figure 2).

**Figure 3:**
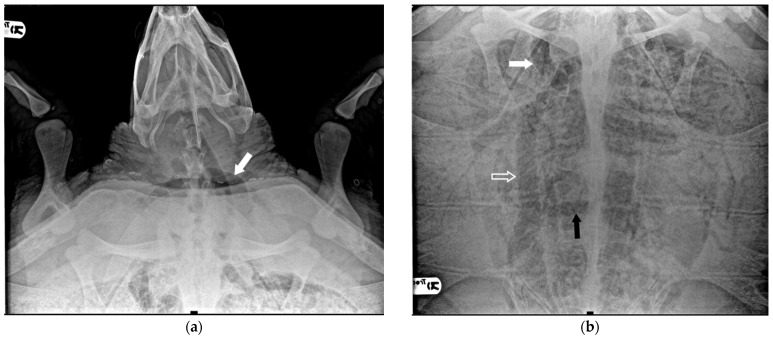
X-ray examination in DV projection of a subject diagnosed with severe GE. An evident massive presence of gas in the majority of vessels: (**a**) the white arrow shows the subclavian artery; (**b**) the white arrow shows the massive presence in the precaval vein, the white empty arrow the postcava vein, the black arrow the transverse vein, obscuring a full view of the lung areas.

Six turtles were categorized as severe (20.7%) because, apart from all the more peripheral districts, a significant presence of gas was observed in central vascular districts such as the precava vein, postcava vein, venous sinus, left atrium, pulmonary artery and vein, pulmonary trunk, aorta, and brachiocephalic trunk (Figure 3).

The ultrasound examination had a duration of 30 to 45 min. The technique was executed without any significant challenges, and the animals consistently displayed good tolerance towards the procedure and the necessary restraint, except for larger turtles, for which dorsal restraint could pose occasional difficulties.

In seven animals (24, 14%), gas presence was not detected, while in 19 cases (65, 52%), gas was confirmed to be present. In three instances (10, 34%) ultrasound exams did not yield a conclusive result. 

**Figure 4:**
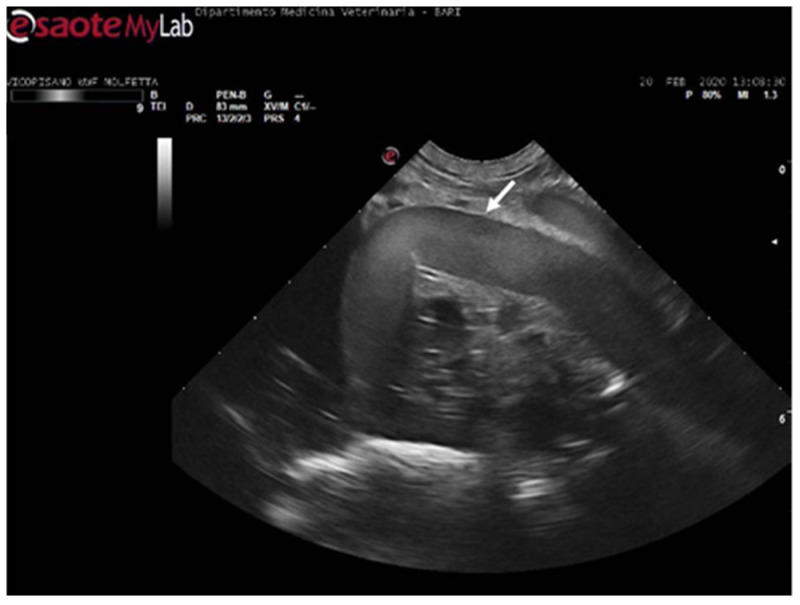
Ultrasound scan from the left prefemoral window reveals normal vascular flow within the iliac artery (the white arrow indicating its wall).

During the ultrasound examination of the seven animals in which no emboli were detected, all the accessible vessels through the acoustic windows were clearly visualized. Blood flow was easily detectable with Color Doppler. From both prefemoral acoustic windows, the renal and iliac arteries were easily distinguishable, along with their blood flow (Figure 4).

From the left prefemoral acoustic window, the liver parenchyma could be distinguished, and the blood flow in the relevant vessels was often observable.

Ultrasonography also provided clear views of the intestinal loops with their vascular structure and the stomach. The ventral cervical window allowed for the distinct visualization of the trachea and esophagus, with their paths easily traceable. No vascular alterations were evident by ultrasound. The same animals had been categorized as negative by the radiological examinations. 

**Figure 5:**
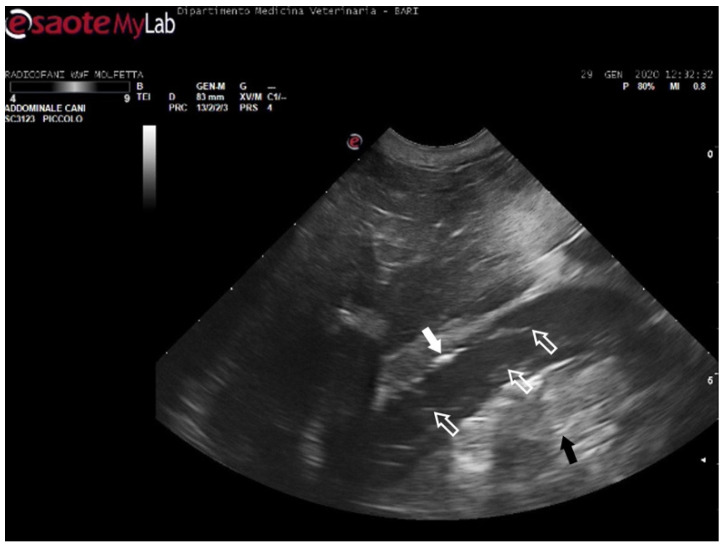
Ultrasound scan from the right prefemoral window shows an aggregate of medium-sized microemboli adhered to the wall of the iliac vein (white arrow), releasing very small emboli into the bloodstream (empty white arrows). The black arrow indicates the renal parenchyma.

In 11 sea turtles (37, 9%), the ultrasound examination revealed the presence of emboli in various districts, often in limited quantity. Those emboli varied in size but were generally considered microemboli. They were observed to enter the bloodstream in a cascade, resulting in slower and intermittent blood flow on Color Doppler ultrasound. Ultrasonography provided distinct visualization of the liver parenchyma and its vessels, allowing for a clear view of the intestinal loops and their associated circulation. The larger emboli were consistently found adhering to the vessel walls, while smaller emboli occasionally detached and flowed into the bloodstream (Figure 5) (Video S1). 

**Figure 6:**
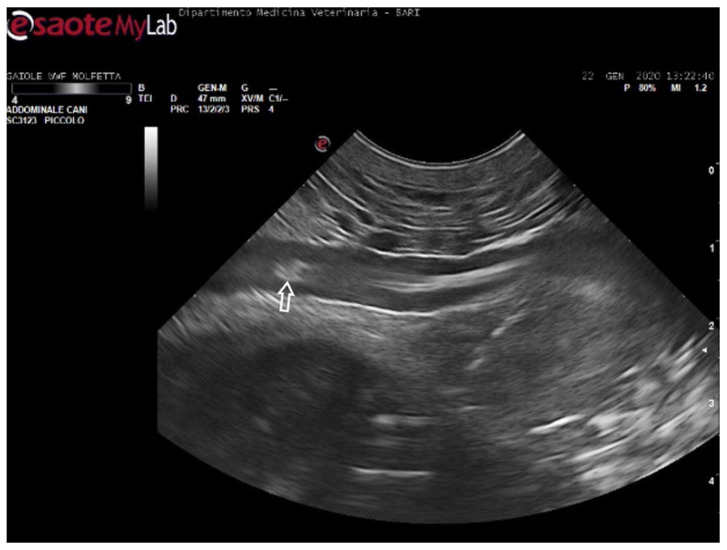
Ultrasound scan from the ventral cervical window revealing an aggregate of sizable microemboli (empty white arrow) in transit within the bloodstream of the left subclavian artery.

The patients displaying these ultrasound findings were placed in the mild category following Parga’s method [17]. In some animals within this group, ultrasonography revealed emboli in multiple areas compared to those identified on radiographs. Additionally, in a turtle that appeared radiographically negative, ultrasound exams detected the presence of microemboli in the renal arteries on both sides, as well as in the left subclavian artery (Figure 6).

**Figure 7:**
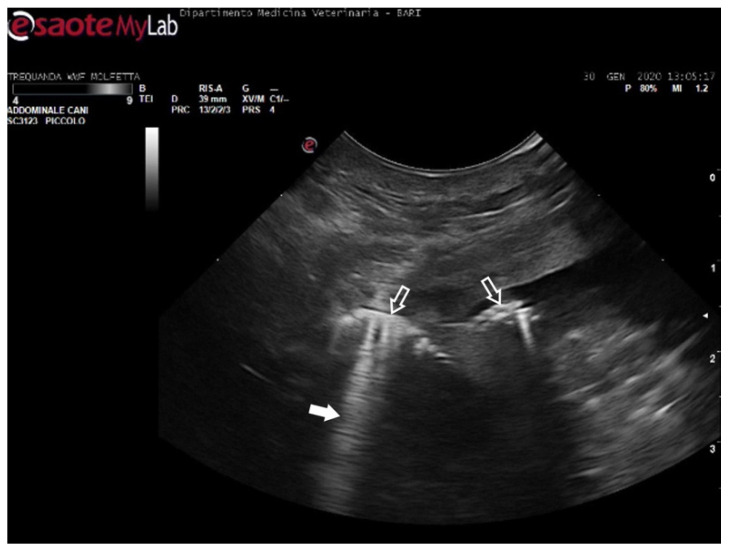
Ultrasound scan from the right prefemoral window revealing several clusters of large emboli attached to the hepatic vessels (empty white arrows). The artifacts generated by their presence (white arrow) make it challenging to visualize deeper structures.

In four turtles (13, 8%), the ultrasound examination revealed a significantly slow blood flow, but no flow was detected using Color Doppler ultrasound. The renal vessels and subclavian vessels, which were closer to the acoustic windows, were easily identified. However, examination of the vessels and the coelomic cavity through the ventral cervical acoustic window was challenging due to significant gas-related disorders. Consequently, visualization of the aortic arch and the heart was precluded. Similarly, it was impossible to locate the mesenteric vessels and differentiate the hepatic parenchyma along with its associated circulation. The presence of larger emboli, some up to 5 mm in size, hindered the visualization of vessel walls, and the gas-induced artifacts, such as comet tails, were so pronounced that they masked the view of tissues and vascular regions (Figure 7) (Video S2). Those ultrasound images have been a consistent finding in the subjects previously categorized by radiographic exams as having moderate GE, implementing Parga’s method [17].

In six turtles (20, 7%), the ultrasound examination revealed a lack of blood flow, where no movement of emboli was detectable. Color Doppler ultrasound detected no flow in these cases. Vascular turbulence caused by the presence of macroemboli was frequent, yet visualization of vessel walls was unattainable. Neither organ parenchyma nor vascular sites could be identified in these cases. In our experience, those ultrasound findings were documented in turtles that have been radiographically classified as having severe GE.

In three large turtles (10, 3%), ultrasonography provided no diagnostic value: radiographically, two of these animals were classified as negative, while one was identified as having mild GE. Consequently, these three patients were excluded from the statistical analysis, which was conducted on a population of 26 sea turtles.

Table 1 presents the comparison of GE severity assessments, based on radiographic and ultrasound findings.

### Statistical Analysis

Considering radiography as the gold standard, a contingency table was constructed to analyze the relationship between variables (Table 2).

In order to determine the “true positive rate”, we calculated the sensitivity index, resulting in 90%. This value indicates a 90% likelihood that a turtle with GE will test positive on an ultrasound examination.

In the same way, to assess the “true negative rate”, we computed the specificity index, resulting in 83.3%. This index confirms an 83.3% likelihood that a turtle without GE will produce a negative result on the ultrasound examination.

In order to evaluate the proportion of correct predictions, including both true positives and true negatives, among all cases examined, we calculated the accuracy index, obtaining 88.46%. In our sample, the ultrasound test effectively determined the turtle’s condition (both healthy and sick) in 88.5% of instances (Table S2).

To estimate the concordance between the two diagnostic procedures, we calculated Cohen’s kappa, which evaluates the concordance index by factoring in the likelihood of random agreement. The value obtained for Cohen’s kappa is 0.69, where the range of 0.61 to 0.80 indicates a good and substantial concordance between the two tests. 

## 4. Discussion

A study involving loggerheads accidentally captured in the Atlantic Ocean explored the use of ultrasonography on board fishing vessels for immediate or early diagnosis of GE [17]. In that study, the severity of GE was classified based on the observed total intravascular gas and its distribution, consistent with the image categorization of García-Párraga et al. [5] for both ultrasound and radiography, as well as Parga’s evaluation [17]. A similar 3-grade scoring system was described in 2021 for radiographic findings by Franchini et al. [18].

Our interest in comparing ultrasonography and radiography (the last one considered as the gold standard test) in diagnosing GE originated from the easier use on board of ultrasound exams, overcoming challenges associated with radiographic exams in such settings. For this purpose, we used a random sample of turtles of different sizes that closely mirrored the actual conditions encountered during fishing trips.

The cardiovascular physiology of sea turtles plays a significant role in the study results. Sea turtles are characterized by an exceptionally slow blood flow, notably distinct from that of mammals, marked by low-frequency and low-intensity cardiac output [19]. Furthermore, the physical properties of blood components in sea turtles have a crucial role in this situation [20]. Notably, the acoustic impedance of the corpuscular blood component is distinctive and highly pronounced, in contrast to mammals, which facilitated the clear delineation of blood flow. This inherent characteristic enabled the precise observation of moving blood in unaffected turtles.

In turtles diagnosed with mild GE, the presence of microemboli was discerned as small hyperechoic spheres in relatively swift motion.

In animals affected by moderate GE, the blood flow exhibited further deceleration, reaching near immobility in subjects with severe manifestations of the disease. It seemed as if substantial quantities of emboli adhering to vessel walls had the capability to dramatically impede blood flow speed.

Among the ultrasound probes tested, the most suitable and high-performing option, chosen for our study, was the broadband multi-frequency Microconvex probe with a 7.5 MHz frequency. This probe offered superior vessel and parenchyma definition. In contrast, the 2.5 MHz broadband multi-frequency linear probe, while capable of concentrating the visual field, resulted in image definition loss, likely due to greater soft tissue interference, and was thus not employed.

Color Doppler imaging provided enhanced visualization of blood flow in negative and mild cases. However, in patients with moderate and severe GE, this technique failed to detect blood flow. Consequently, while Color Doppler has proven ineffective for flow localization, it has been valuable in assessing the severity of GE. 

The slowness and intermittency of circulatory flow, a hallmark of these animals, necessitated relatively extended ultrasound examination times and patience by operators and animals [21]. To form a conclusive judgment regarding the presence or absence of emboli in the bloodstream, it was always crucial to await moments coinciding with slow cardiac contractions or synchronized with respiratory cycles, during which circulation accelerated.

The standardized technique employed, providing the visualization of all the acoustic windows, revealed the presence of microemboli even in turtles which tested negative on radiographic examination. Additionally, in subjects with mild severity, it confirmed the presence of gas in multiple areas compared to those identified via radiographic imaging. Based on these findings, we propose that observation should not be restricted to renal vessels only, and it is advantageous to regularly assess all available districts.

In turtles affected by moderate GE, ultrasound imaging was often unclear due to the presence of artifacts caused by substantial bubble accumulation. These artifacts impeded visualization of intracoelomic viscera and associated circulation. Only the most superficial vessel districts, particularly those closer to the acoustic windows, could be identified, such as renal and subclavian vessels. In these areas, large macroemboli were occasionally observed, typically adhering to the vessel walls. This phenomenon may be attributed to subcutaneous and muscular tissues surrounding the acoustic windows not retaining gas, unlike organ parenchyma. This is inundated with copious gas in cases of moderate GE.

Conversely, observations differed when examining subjects with severe manifestations of the disease. In such cases, the presence of a significant amount of gas made exploration of the coelomic cavity impractical, due to the generation of numerous substantial artifacts. The absence of blood flow was the predominant feature observed, hindering the identification of vessel walls. 

Drawing from our experience with the cases examined, we think that relying solely on ultrasound findings may not be sufficient for evaluating GE, as it may not always be achievable through ultrasonography alone. Ultrasonography was non-diagnostic in the three largest subjects (approximately 50 kg) as the target organs and vascular districts could not be reached via any acoustic window, consistent with the existing literature [11,17]. For this reason, these animals were excluded from our statistical analysis. 

Ultrasonography was valuable in identifying the disease even in a case where radiography showed negative results, and in detecting gas in anatomical sites not visible on radiography in mild cases. However, it is important to note the extensive time required for the examination and its diagnostic limitations in cases involving overly large animals or severe GE.

The detection of gas by ultrasonography, in cases where radiography shows negative results, may be attributed to the timing of the gas-off phase in affected turtles. In the early stages of this phase, microemboli of intravascular gas may be dispersed throughout the body, potentially leading to an overestimated GE scoring when assessed solely by ultrasonography [17].

In cases of negative radiographic results, ultrasonography demonstrated a good diagnostic value, but its longer execution time in comparison to radiography limits its use primarily to situations where radiography is not feasible.

## 5. Conclusions

The results of this study indicate that the combined use of ultrasonography and radiography is valuable for evaluating GE. Further research with repeated ultrasound examinations at various stages of disease progression is desirable to validate its diagnostic efficacy. The results of this study indicate strong and statistically significant concordance between radiographic and ultrasound findings. Our experience suggests that, while ultrasound examination cannot completely replace radiographic evaluations, the combination of both techniques provides greater precision in the evaluation of the severity of GE.

## Figures and Tables

**Table 1:** Comparison of the radiographic and ultrasound findings, including severity assessment as determined by both radiography and ultrasonography.

ID Code	XR	Degree by XR	US	Degree by US
102213	Positive	Severe	Positive	Severe
102037	Negative	Mild	Undetectable	ND
102099	Positive	Mild	Undetectable	ND
102097	Negative	Negative	Undetectable	ND
102146	Positive	Mild	Negative	Mild
102156	Positive	Mild	Positive	Mild
102131	Positive	Moderate	Positive	Moderate
102038	Positive	Mild	Positive	Mild
102231	Positive	Mild	Positive	Mild
102143	Positive	Mild	Positive	Mild
102098	Negative	Negative	Negative	Negative
102106	Negative	Negative	Negative	Negative
102280	Negative	Negative	Negative	Negative
102229	Positive	Moderate	Positive	Moderate
102147	Positive	Mild	Positive	Mild
102162	Positive	Moderate	Positive	Moderate
102216	Positive	Severe	Positive	Severe
102192	Positive	Moderate	Positive	Moderate
102152	Positive	Mild	Positive	Mild
102158	Positive	Mild	Positive	Mild
102160	Positive	Severe	Positive	Severe
102224	Positive	Mild	Positive	Mild
102212	Negative	Negative	Positive	Negative
102127	Positive	Mild	Positive	Moderate
102026	Positive	Mild	Negative	Mild
102161	Positive	Moderate	Positive	Moderate
102125	Negative	Negative	Negative	Negative
102159	Positive	Severe	Positive	Moderate
102215	Negative	Negative	Negative	Negative

**Table 2:** Contingency table with radiography as the gold standard test.

	XR Positive GE	XR Negative GE	
US visible emboli	18 (a)	1 (b)	19 (visible emboli by US)
US no visible emboli	2 (c)	5 (d)	7 (no visible emboli by US)
	20 (visible emboli by RX)	6 (no visible emboli by RX)	26 loggerheads

(a) indicates the 18 positive cases for GE with radiology and ultrasound; (b) indicates 1 negative case for GE with radiology but positive for ultrasound; (c) indicates 2 positive cases for GE with radiology but negative with ultrasound; (d) indicates 5 negative cases for GE with radiology and ultrasound.

## Data Availability

The data presented in this study are available on request from the corresponding author.

## Supplementary Materials

The following supporting information can be downloaded at: https://www.mdpi.com/article/10.3390/ani14243623/s1. Table S1: Morfometric measurement including the CCL, CCW and weight; Table S2: Comparision of ultrasonography performance with radiography as the gold standard test, based on the contingency table; Video S1: Ultrasound examination of a subject with mild GE: within the normal flow pattern, sporadic emboli are observed. The flow remains normal, and the microemboli do not generate artifacts; Video S2: Ultrasound examination of a subject with moderate GE: the flow is slowed, and larger mobile emboli are observed, causing artifacts.
