# Supplementary material for: Comparative Diagnostic Efficacy of Ultrasonography and Radiography for Gas Embolism in Loggerhead (Caretta caretta) Turtles
Source: Animals (Basel). 2024 Dec 16;14(24):3623. doi: 10.3390/ani14243623 (PMC11672709; doi:10.3390/ani14243623)
Supplement: Supplementary file 1 [file animals-14-03623-s001.zip › Table S1 and S2.pdf]

Table S1. Morphometric measurement including the Curved Carapace Lengh (CCL), Curved Carapace Width (CCW) and Weight.

ID Code	CCL (cm)	CCW (cm)	Weight (kg)
102213	66.9	58.5	36.9
102037	75.4	67	51.5
102099	75.9	67.2	48
102097	75.8	69.5	56.8
102146	71.5	66.4	44.9
102156	66	58.5	32.7
102131	65.5	63	42.2
102038	70.7	64.8	40.8
102231	66	59.5	33.2
102143	62.6	57.4	28.3
102098	28.1	26.5	2.7
102106	75.9	67.3	19.35
102280	67.4	61.8	31.2
102229	68	62	40.1
102147	62	57	28.1
102162	56.5	53.5	21.8
102216	63	57.5	28.9
102192	57	49.8	21.1
102152	71	64.5	43
102158	63	57	29
102160	70.5	62	43.2
102224	58.9	54	24.6
102212	71.2	62.8	40.3
102127	73.4	69.2	52.2
102026	66.6	57	32
102161	71.5	67.6	40.2
102125	60.2	57.1	24.7
102159	68.5	61.5	59.5
102215	50	46.4	16.65

Table S2. Comparison of ultrasonography performance with radiography as the gold standard test, based on the contingency table:

Sensitivity = a / a+c = 18/20 = 0.90= 90%

Specificity = d / b+d= 5/6 = 0.8333= 83.3%

Accuracy = a+d / a+b+c+d = 23/26 = 0.8846 = 88.46

Video S1. Ultrasound examination of a subject with mild GE: within the normal flow pattern, sporadic emboli are observed. The flow remains normal, and the microemboli do not generate artifacts.

Video S2. Ultrasound examination of a subject with moderate GE: the flow is slowed, and larger mobile emboli are observed, causing artifacts.
